# Dietary Therapies for Gastrointestinal Disorders

**DOI:** 10.3390/nu18111787

**Published:** 2026-06-01

**Authors:** Berkeley N. Limketkai, Andrea Shin, Natalie Manitius, Sameeha Rau, Janelle Smith, Neha D. Shah

**Affiliations:** 1 Division of Digestive Diseases, UCLA School of Medicine, Los Angeles, CA 90095, USA; 2 Division of Clinical Nutrition, UCLA School of Medicine, Los Angeles, CA 90095, USA; 3 Salvo Health, New York, NY 10017, USA; 4 Division of Gastroenterology, UCSF School of Medicine, San Francisco, CA 94115, USA

**Keywords:** diet, nutrition, medical nutrition therapy, irritable bowel syndrome, Crohn’s disease, ulcerative colitis, functional dyspepsia, constipation, diarrhea

## Abstract

Alterations in gastrointestinal function (digestion, absorption, motility, secretion, and elimination) play important roles in the pathophysiology of many gastrointestinal disorders. Food also strongly influences gastrointestinal health and disease. Some foods act as antigens that trigger an enteric immune response, while others can serve as substrates with direct or indirect biological effects. Food can also be metabolized by gut microbes into bioactive molecules that alter physiology. This review discusses the current research evidence and the clinical use of “food as medicine” through dietary therapies for the management of various gastrointestinal conditions, including disorders of gut–brain interaction, eosinophilic esophagitis, celiac disease, inflammatory bowel disease, gastroparesis, and short bowel syndrome with intestinal failure.

## 1. Introduction

Besides serving as essential nutrients for the body’s maintenance and growth of life, food has direct and indirect effects on biological function that can worsen or ameliorate disease. For one, food can serve as an antigen that directly provokes an enteric immunologic response, such as in the cases of celiac disease and allergic food hypersensitivities [1,2]. Other nutrients may possess intrinsic antioxidant, anti-inflammatory, and antimicrobial properties [3]. Food can also be metabolized by gut bacteria into bioactive molecules that invoke physiologic effects [4]. Alternatively, the post-absorption processing of nutrients can lead to functional downstream metabolites, such as in the conversion of omega-6 polyunsaturated fatty acids into pro-inflammatory prostaglandins and leukotrienes along the arachidonic acid pathway [5]. Furthermore, food can trigger gastrointestinal symptoms in individuals with underlying intolerance or malabsorption. These significant effects on human physiology have contributed to a growing interest in “food as medicine”.

Among organ systems, the gastrointestinal tract has the closest relationship with food; its central function is the digestion and absorption of nutrients, electrolytes, and water. As such, the health and function of the digestive system can be strongly influenced by the foods we eat. This narrative review discusses the available research and clinical application of dietary therapies for the management of various gastrointestinal conditions: disorders of gut–brain interaction (DGBI), eosinophilic esophagitis (EoE), celiac disease (CeD), inflammatory bowel disease (IBD), gastroparesis, short bowel syndrome (SBS) and intestinal failure. These disorders were selected since they represent a spectrum of gastrointestinal disorders across functional, inflammatory, and malabsorptive mechanisms. Moreover, diet plays a central role in their underlying pathophysiology, symptom development, and evidence-based management. For some other disorders, such as peptic ulcers, gastroesophageal reflux disease, pancreatitis, and gastrointestinal cancers, nutrition remains an important facet of disease management but serves a more adjunctive role and/or has less supporting research data.

## 2. Disorders of Gut–Brain Interaction

Symptom exacerbation associated with dietary intake is commonly reported by patients with DGBI, including irritable bowel syndrome (IBS) and functional dyspepsia (FD) [6,7,8]. Meal-related symptoms are further embedded within the diagnostic criteria for postprandial distress syndrome, a subtype of FD. Consequently, individuals with DGBI may be susceptible to maladaptive eating behaviors that can lead to suboptimal nutritional intake, underscoring the importance of understanding the role of diet in the pathophysiology and management of these disorders.

Although the precise cause of dietary sensitivities and intolerances are often difficult to define at the individual level, multiple lines of evidence support the involvement of overlapping mechanisms involving luminal, mucosal, and central pathways. Luminal distension and altered intestinal transit, driven by the osmotic effects and gas production resulting from microbial fermentation of dietary substrates—such as fermentable oligosaccharides, disaccharides and monosaccharides, and polyols (FODMAPs)—may contribute to symptoms in IBS and have also been implicated in FD [9,10]. In parallel, accumulating evidence supports a role for food-induced immune activation, whereby the disruption of the intestinal barrier in response to food antigens or neuroactive metabolites generated through the microbial fermentation of food may promote immune responses, including eosinophil and mast cell activation—particularly in the context of stress or infection—leading to visceral hypersensitivity and food-related symptoms [11,12]. In addition, altered gastrointestinal sensorimotor functions may play an important role, particularly in FD, where impaired gastric accommodation, delayed gastric emptying, and heightened sensitivity to gastric or duodenal distention contribute to early satiety, fullness, and discomfort following meals [13]. Finally, central factors, including psychological stress, anxiety, hypervigilance, and learned expectations may interact with peripheral mechanisms to amplify symptoms or reinforce food intolerances in DGBI, emphasizing the complex bidirectional gut–brain interactions that shape the patient experience [8,14].

**Figure 1:**
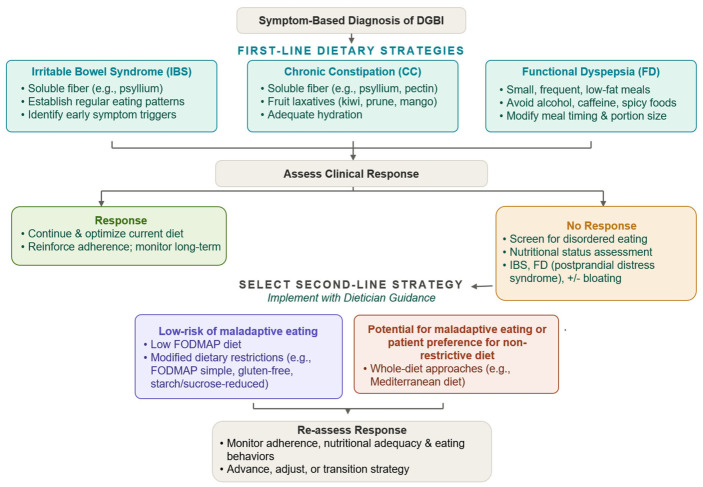
Algorithm of dietary therapies for disorders of gut–brain interaction.

Dietary therapies are commonly recommended as a first-line strategy in DGBI management (Figure 1). Although the strongest evidence for the efficacy of dietary therapies exists for IBS, dietary modifications are often employed for many other DGBI, such as chronic constipation (CC) and FD. Recognizing that most pharmacological treatments improve symptoms in less than 50% of patients, dietary therapies play a crucial part in the multimodal approach to patient care [15]. These principles may also extend to other overlapping DGBI that often coexist or occur along a clinical continuum with IBS [16,17].

### 2.1. Dietary Therapies for Bowel Disorders of Gut–Brain Interaction

Initial dietary therapies for bowel disorders, such as IBS and CC, often focus on increasing fiber intake. Current guidelines endorse soluble fiber, but not insoluble fiber, for improving global IBS symptoms [18]. These recommendations are supported by a meta-analysis of 15 randomized controlled trials (RCTs) which demonstrated that fiber reduced the risk of remaining symptomatic in IBS (relative risk [RR] 0.86; 95% confidence interval [CI] 0.80–0.94), with benefits limited to soluble fiber; bran, an insoluble fiber, showed no effect [19]. Similarly, evidence for soluble fiber supplementation in CC was demonstrated in a meta-analysis of 16 RCTs, in which fiber was associated with symptomatic improvement (RR 1.48; 95% CI 1.17–1.88). Benefits were primarily observed with soluble fibers, including psyllium and pectin [20]. Fruit-based laxatives, such as kiwifruits, mangos, and prunes, may also be considered for CC due to their natural fiber content and osmotic effects [21].

Following adjustments in diet and eating patterns, a substantial body of evidence supports the use of second-line dietary therapies in IBS, including short-term elimination diets, such as those low in FODMAPs, gluten, starch, or sucrose, as well as whole-food diets, such as the Mediterranean diet [22]. When implementing elimination diets, careful patient selection is essential to identify individuals who may benefit from more intensive dietary modification and are at a low risk for maladaptive or disordered eating; such dietary therapies should ideally be administered with the guidance of a registered dietitian [15].

In a recent systematic review and network meta-analysis, a low FODMAP diet was superior to a habitual diet for global IBS symptoms (RR 0.51; 95% CI 0.37–0.70), abdominal pain (RR 0.61; 95% CI 0.42–0.89), and bloating (RR 0.55; 95% CI 0.37–0.80), but not bowel habits [23]. Other approaches, including starch- and sucrose-reduced, gluten-free, and FODMAP “simple” diets, also ranked highly and were superior to a habitual diet for global IBS symptoms, suggesting that less restrictive diets may also be effective for some individuals. Notably, most dietary trials have been conducted in patients with non-constipated IBS. Evidence for the role of restrictive dietary interventions in non-painful bowel DGBI, such as CC, is lacking [22].

IgG-based elimination diets have recently been evaluated as a novel approach for guiding targeted dietary changes in IBS. In a recent multi-center RCT, IgG-based elimination diets were associated with significantly higher response rates for abdominal pain compared to sham diets, particularly among those with constipation-predominant IBS or mixed bowel habits [24]. However, it remains unclear whether food-specific IgG levels reflect the true pathophysiological, immune-mediated mechanisms of food sensitivities or merely a response to dietary exposures (i.e., an epiphenomenon). Moreover, IgG-based testing frequently implicates wheat and milk as triggers, raising the possibility that the efficacy of these diets may result from the elimination of typical sources of food intolerances rather than immune-mediated processes. Given these limitations, further validation is required before IgG-based testing can be recommended for routine clinical use.

Despite their potential efficacy, elimination diets can be challenging to implement and may adversely affect psychological and social well-being. Accordingly, the feasibility and acceptability of dietary therapies are critical considerations in clinical practice [25]. Reintroduction trials are essential to identify specific dietary triggers while minimizing unnecessary long-term dietary restrictions, thereby helping to preserve nutritional adequacy, dietary diversity, and quality of life. In this context, recent studies evaluating the efficacy of less restrictive diets, such as the Mediterranean diet, have shown promise in improving gastrointestinal symptoms and abdominal pain in IBS [26,27,28]. The utility of combining the low FODMAP and Mediterranean diets has also recently been tested in an RCT, which demonstrated superiority with a combined diet over standard dietary advice; however, whether this strategy offers additional benefit beyond either the low FODMAP or Mediterranean diet alone remains to be determined [29].

### 2.2. Dietary Therapies for Functional Dyspepsia

First-line dietary therapies for FD frequently emphasize meal pattern modification rather than the increased intake of specific nutrients or foods. Strategies may include consuming smaller, more frequent meals with low-fat content and avoiding potential triggers, such as alcohol, caffeine, and spicy foods; however, the benefits of these modifications have not been systematically assessed in RCTs [30,31]. Only a few clinical trials have evaluated the efficacy of elimination diets in patients with FD.

Beyond general dietary modifications, evidence supporting structured dietary therapies for FD remains limited. In one randomized study comparing the low- FODMAP diet with traditional dietary advice, there were no significant differences in symptoms between groups. However, in subgroup analyses, significant symptom responses were observed in patients with postprandial distress syndrome or bloating to potentially indicate that responses may vary according to FD subtype [32]. In another open-label study, the effects of FODMAP restriction followed by a blinded reintroduction on the symptoms were assessed in 36 patients with postprandial distress syndrome to demonstrate an improvement in the majority (73%) of patients [33]. Duodenal biopsies were obtained to evaluate the mucosal integrity at baseline and after six weeks of FODMAP restriction, but no significant changes were seen. The reintroduction of FODMAP powders demonstrated mannitol to be the most common FODMAP trigger, which has also been reported in reintroduction trials conducted among patients with IBS [34]. Although findings indicate a potential role for the low- FODMAP diet in FD, the research is limited. Whether the observed benefits reflect responses due to comorbid or overlapping IBS have yet to be determined.

### 2.3. Summary

Diet plays a central but complex role in the pathophysiology and management of DGBI, with converging evidence supporting contributions from luminal, immune, sensorimotor, and central mechanisms. Dietary therapies are a foundational component of DGBI, with the strongest evidence in IBS, while the research in FD and other DGBI remains more limited (Table 1). Although elimination diets, such as the low- FODMAP diet, may be effective for many patients, concerns regarding the long-term sustainability, nutritional adequacy, and potential impacts on eating behaviors highlight the need for careful patient selection and a structured implementation under the guidance of a registered dietitian. Emerging evidence for the use of less restrictive whole-diet approaches suggests that a shift towards these strategies may be feasible and effective for some individuals, potentially reducing the burden associated with strict elimination protocols. Future research should focus on several key priorities: (i) identifying the predictors of the dietary response; (ii) clarifying the mechanisms of food-related symptoms across the spectrum of DGBI; (iii) expanding high-quality trials to DGBI beyond IBS; (iv) evaluating long-term outcomes including adherence and the nutritional adequacy of dietary therapies; and (v) developing simplified and/or less restrictive diets to minimize patient burden.

## 3. Eosinophilic Esophagitis

EoE is a chronic immune-mediated, inflammatory disorder of the esophagus affecting both adults and children. It is characterized by symptoms of esophageal dysfunction, such as dysphagia, food impaction, vomiting, regurgitation, and failure to thrive. The pathophysiology is complex and involves genetic, environmental, and immunologic factors. The exposure to certain food allergens triggers the release of type 2 cytokines (i.e., interleukin [IL]-4, IL-5, and IL-13) from type 2 helper T-cells with the resultant activation and recruitment of eosinophils to the esophagus. This inflammatory cascade leads to esophageal fibrosis and dysfunction [35,36]. EoE is more common in white males and those with a history of atopy; notably, 50% of adults initially presenting with a food impaction are ultimately found to have EoE [37]. According to the American College of Gastroenterology (ACG) clinical practice guidelines, the diagnosis of EoE includes symptoms of esophageal dysfunction and at least 15 eosinophils per high-power field (eos/hpf) on the esophageal biopsy among six samples from at least two esophageal regions (proximal/mid and distal) [38]. Typical endoscopic features include linear furrows, rings, or strictures. Management focuses on reducing esophageal inflammation with proton pump inhibitors, topical corticosteroids, or dietary therapy. For those who do not respond, dupilumab (IL-4 and IL-13 inhibitor) is a second-line option. Patients with evidence of fibrostenotic strictures may also require endoscopic dilation in parallel with anti-inflammatory therapy.

### 3.1. Dietary Therapies for Eosinophilic Esophagitis

Dietary therapies for EoE include exclusive enteral nutrition (EEN) and empiric elimination diets, both of which aim to reduce esophageal inflammation by removing or minimizing the exposure to food antigens.

#### 3.1.1. Enteral Nutrition

Diet was first recognized to play a role in esophageal inflammation in a 1995 landmark study by Kelly et al. [39] The authors found that patients with presumed gastroesophageal reflux disease (GERD) refractory to anti-reflux therapies and with increased esophageal eosinophils experienced an improvement in symptoms and a reduction in mucosal eosinophils after treatment with an elemental (predigested) diet using an amino-acid based, hypoallergenic formula exclusively. A subsequent clinical trial in 2003 by Markowitz et al. demonstrated that 49 out of 51 (96%) children and adolescents with EoE treated with an elemental diet for one month had significant improvement in vomiting, abdominal pain, and dysphagia, as well as a significant reduction in the median number of eos/hpf (33.7 to 1.0; *p* < 0.01) after dietary therapy [40]. Similarly, a 2013 study by Peterson et al. with 18 adults demonstrated a significant reduction in mucosal eosinophils after treatment with an elemental diet for 2–4 weeks (54 to 10; *p* < 0.01) [41]. However, the noncompliance/dropout rate was high at 38%.

Despite its efficacy, EEN has limits with real-world utility. In the study by Markowitz et al., most pediatric patients were unable to drink the formula orally due to poor palatability and opted for nasogastric administration instead. Elemental formulae are also expensive and not universally reimbursed by insurance companies. Furthermore, the dietary reintroduction phase is complex and prolonged, requiring serial endoscopies to ensure sustained histologic remission, further limiting feasibility in clinical practice.

#### 3.1.2. Food-Allergy-Guided Elimination Diet

Given the practical limitations associated with EEN, there was interest in using allergy testing, such as skin prick, atopy patch, and/or serum IgE testing, to guide dietary therapy for EoE. A systematic review of 16 studies found that allergy-test-driven diets were effective in 66% of cases and not superior to empiric elimination diets [42]. Similarly, a study involving 50 adults with EoE demonstrated that skin-prick testing predicted only 13% of foods associated with EoE [43]. Since allergy testing has a low predictive value for EoE triggers, it is not recommended by the ACG to guide food elimination diets [38]. Instead, current guidelines by the ACG recommend empiric elimination diets that remove the most common allergens known to trigger EoE.

#### 3.1.3. Six-Food Elimination Diet

**Figure 2:**
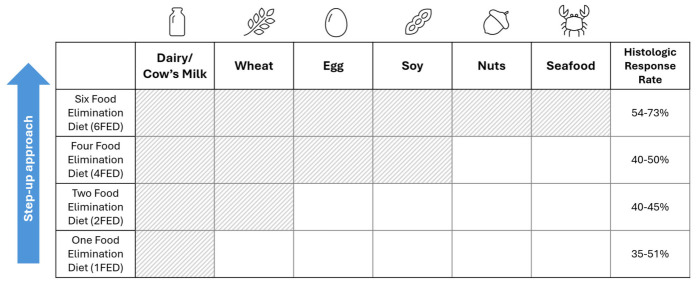
Composition of elimination diets for eosinophilic esophagitis.

The six-food elimination diet (6FED) involves the avoidance of dairy, soy, eggs, fish/shellfish, nuts, and wheat for 6–8 weeks, followed by sequential reintroduction and serial endoscopic assessments to identify specific trigger foods (Figure 2). Several studies have demonstrated the efficacy of the 6FED in inducing both clinical and histologic remission. A retrospective study in 2006 found that 74% of patients treated with 6FED had a significant improvement in eos/hpf, compared with 88% of patients treated with an elemental diet [44]. These results suggest a similar efficacy of 6FED and elemental diets; however, 6FED is associated with a lower cost, improved palatability, and greater adherence to treatment. A prospective single-center study showed a similar significant reduction in mean eos/hpf on proximal esophageal biopsies from 34 to 8 after treatment with 6FED [43]. Milk and wheat were most frequently associated with EoE recurrence during the reintroduction phase. Another prospective single-center study including 67 adults showed that 73% of patients treated with 6FED achieved a histologic response (defined as peak count < 15 eos/hpf) which was maintained for up to 3 years with the continued avoidance of the identified trigger food(s) [45].

#### 3.1.4. Four-Food Elimination Diet

Although effective, the 6FED is quite restrictive for patients and, after sequential reintroduction and serial endoscopic assessments, typically only one to two food groups are identified as triggers for the majority of patients [45,46,47]. To improve adherence rates and minimize the number of endoscopies needed to identify food triggers, the four-food elimination diet (4FED) was proposed, in which dairy, wheat, soy, and eggs are eliminated. A 2014 prospective, multicenter study showed that 54% of patients achieved clinicopathologic remission with the 4FED [48]. In patients who did not respond to the 4FED, escalation to a rescue 6FED achieved remission in one-third of patients who failed the 4FED, with a 72% remission rate overall.

#### 3.1.5. Two-Food Elimination Diet

Building on these results, the step-up empiric elimination diet was created, in which patients began with a two-food elimination diet (2FED), where the most common food triggers, dairy and wheat, are eliminated for 6–8 weeks. If histologic remission was not achieved, then patients would “step-up” to the 4FED, followed by 6FED if needed. A 2018 study by Molina-Infante et al. assessed the efficacy of the step-up empiric elimination diet in adult and pediatric patients [46]. Forty-three percent of patients achieved remission with the 2FED. Non-responders were offered to step-up to a 4FED, followed by 6FED, with remission rates of 60% and 79%, respectively. The authors also found that a step-up approach reduced endoscopic procedures and diagnostic process time by 20%.

The ACG guidelines recommend that elimination diets for EoE should be done with the following considerations: (1) after ruling out other causes of elevated esophageal eosinophils; (2) with shared decision-making between the provider and patient, given the long-term adherence necessary; (3) not done in conjunction with pharmacotherapy; (4) with guidance from a specialized gastrointestinal registered dietitian; (5) with follow-up endoscopies to evaluate the response, rather than rely on the symptoms alone; and (6) in conjunction with feeding therapy or integrative support in cases of weight loss, fear of food, and disordered eating. The elimination diet should always be followed by a reintroduction phase involving the addition of a food or food group for 6–8 weeks; an assessment of the symptomatic and histologic effect is performed via upper endoscopy. Symptom recurrence or an increase in eosinophils to ≥15 eos/hpf suggests the reintroduced food is a trigger.

#### 3.1.6. One-Food Elimination Diet

Kliewer et al. conducted an RCT comparing the 6FED with the one-food elimination diet (1FED), in which animal milk is excluded, in 129 adult patients [49]. There was no significant difference in the histologic remission rate (defined as peak count of <15 eos/hpf) between the two groups (40% for 6FED vs. 34% for 1FED, *p* = 0.58). However, the 6FED group had a higher rate of “complete remission” (defined as peak count ≤ 1 eos/hpf; 19% vs. 6%, *p* = 0.031). Kliewer et al. also performed an RCT comparing the 1FED versus 4FED in pediatric patients and found similar histologic remission rates between the groups (41 vs. 44%, *p* = 1), though there was a greater improvement in symptom score in the 4FED group (−25 vs. −14.5, *p* = 0.04) [50]. A meta-analysis compared the efficacy of the 6FED, 4FED, 1FED, and a targeted elimination diet (TED) guided by allergy testing in adult and pediatric patients. While the 6FED was most efficacious in achieving histologic remission (61%) compared with the 4FED (49%), 1FED (51%), and TED (46%), this difference was not statistically significant [51]. Together, these studies highlight the potential for less restrictive elimination diets to be used as the initial dietary therapy for EoE management.

### 3.2. Summary

Dietary therapies play a critical role in the management of EoE and, in fact, are the only treatment modality targeting the underlying mechanism of the disease, potentially obviating the need for long-term pharmacologic therapy (Table 2). Although most of the evidence supports the use of 6FED, emerging data suggests that less restrictive diets may yield similar results while improving palatability and reducing the amount of time and number of endoscopy assessments required to identify food triggers. The ACG recommends empiric food elimination for the treatment of EoE, but the overall quality of the evidence remains low, given the lack of placebo-controlled trials and heterogeneity among the studies [38]. Additional studies are needed to compare dietary therapies, define a standardized protocol for food reintroduction, and examine the psychosocial and financial impact of restrictive diets and serial endoscopic evaluations. In the meantime, a 6-to-8-week timeframe for symptoms and endoscopic evaluation may be appropriate prior to the escalation of elimination diets in an empiric step-up approach [46].

## 4. Celiac Disease

CeD is a chronic, immune-mediated enteropathy triggered by dietary gluten in genetically predisposed individuals affecting approximately 1% of the global population and is the most common cause of malabsorption in Western countries. Clinical presentations range from classic diarrhea and wasting to subtler patterns of nutrient deficiency, fatigue, anemia, bone loss, neuropathy, migraines, ataxia, and infertility [1]. Following ingestion, gluten-derived peptides from wheat, barley, and rye resist complete enzymatic digestion in humans. The resulting partially degraded peptides cross the intestinal epithelium and undergo deamidation by the tissue transglutaminase enzyme. In individuals with CeD, the modified peptides are then presented by HLA-DQ2 or HLA-DQ8 molecules on antigen-presenting cells to CD4+ T cells, initiating a Th2-driven inflammatory cascade, leading to villous atrophy, crypt hyperplasia, and increased intraepithelial lymphocyte infiltration in the proximal small intestine. This mucosal damage directly impairs nutrient absorption, particularly of iron, calcium, folate, fat-soluble vitamins, and zinc [52].

### 4.1. Dietary Therapies for Celiac Disease

As the disease mechanism is antigen-driven, the sole treatment of CeD is a lifelong strict adherence to the gluten-free diet (GFD), requiring the complete elimination of dietary wheat, barley, and rye, as well as the prevention of cross-contamination. The GFD resolves symptoms and promotes mucosal healing in most patients with CeD. Symptoms and serologic improvement may occur within months, but full mucosal recovery in adults can take up to three years [1]. However, strict dietary compliance is reported by only 42% to 91% of patients depending on the assessment method used, and up to half of patients who adhere well to the GFD may continue to display villous atrophy [53].

The GFD is frequently associated with multiple nutritional imbalances [54]. As an example, fiber intake is consistently lower in celiac patients compared with the general population, partly because many high-fiber grains are excluded by the diet and partly because processed gluten-free products are typically made from low-fiber starches [55]. This may be counteracted by increasing naturally gluten-free foods in the diet, including legumes, buckwheat, and labeled gluten-free whole grains, rather than relying on processed substitutes [56]. Individuals with CeD should receive a systematic nutritional assessment and monitoring from the point of diagnosis, centering the role of the specialized gastrointestinal registered dietitian (Table 3) [55].

The adherence to the GFD can also be suboptimal due to intentional and unintentional gluten ingestion. Unintentional gluten cross-contact may occur during food cultivation, processing, and preparation. Patients with CeD require education on how to avoid gluten by reading food ingredient labels and dietary claims, how to physically separate gluten in domestic kitchens, and what questions to ask when dining out or consuming commercially prepared foods [57].

Foods and grains that are naturally gluten-free may contain gluten via cross-contact in cultivation and milling, particularly oats [58]. Research on gluten cross-contact in both domestic and commercial kitchens is in its infancy, and as such evidence-based guidelines are not available. Potential sources of cross-contact with gluten in food preparation include airborne flour particles, shared cooking equipment, and shared use of condiments, cooking oil, and cooking water [57,59].

Quality of life is also significantly impaired on the GFD, reflecting an impact on psychosocial function. Quality of life is especially known to reduce adherence in cases of food insecurity, environmental challenges, and lack of social support [60,61]. In some, hypervigilance to the GFD is also associated with comorbid psychiatric conditions, including eating disorders, requiring multidisciplinary support from psychologists and specialized registered dietitians [62,63].

### 4.2. Non-Responsive and Refractory Celiac Disease

A substantial proportion of patients with CeD continue to experience symptoms despite following a GFD, a situation termed non-responsive celiac disease (NRCD). NRCD is “relatively common” and most frequently caused by inadvertent gluten ingestion, which often goes unrecognized by the patient [64]. NRCD should be evaluated with a comprehensive dietary review, celiac autoantibody serologic testing, a detailed dietary history with an experienced dietitian, and repeat endoscopic biopsies [65]. The dietitian is “an indispensable member of the healthcare team” for patients with NRCD, performing a function that is both therapeutic and diagnostic, since a thorough dietary review often prevents unnecessary invasive workups or premature referral to secondary care [66]. After gluten exposure has been excluded, the clinician must systematically evaluate the patient for other causes of persistent symptoms, such as IBS, microscopic colitis, pancreatic exocrine insufficiency, lactose or fructose intolerance, and small intestinal bacterial overgrowth. In many cases, a low FODMAP diet may improve persistent symptoms from CeD despite the good GFD adherence [67].

Refractory celiac disease (RCD) affects roughly 1% of patients, defined by persistent symptoms and villous atrophy despite at least 12 months of a verified strict GFD. RCD is divided into two subtypes: type I features a normal intraepithelial lymphocyte population and generally carries a more favorable prognosis; type II is characterized by aberrant, clonal T-cell populations in the intestinal epithelium and carries a significant risk of progression to enteropathy-associated T-cell lymphoma [64]. Patients with RCD-II and its lymphomatous complications frequently develop severe protein-calorie malnutrition, nutrient deficiencies, weight loss, and hypoalbuminemia due to profound malabsorption combined with the systemic catabolism. A detailed nutritional assessment is essential in RCD, with an investigation of the micronutrient and macronutrient deficiencies and identified serum albumin as an independent prognostic factor [65]. Oral supplementation to correct the deficiencies should initially be attempted. Patients with more significant malnutrition may require enteral support via nasogastric or nasojejunal tubes for short-term feeding, or gastrostomy tubes for longer-term management. In cases of severe malabsorption where enteral delivery is insufficient, parenteral nutrition (PN) becomes necessary to correct deficiencies and reverse catabolism.

### 4.3. Summary

The GFD remains the primary treatment for CeD to resolve symptoms and promote mucosal healing in most patients, and to reduce the risk of long-term complications, including lymphoma. However, the GFD is not nutritionally complete on its own, and the notion that “going gluten-free” constitutes an adequate treatment without ongoing nutritional monitoring is outdated and potentially harmful. Research consistently shows that macronutrient imbalances are associated with processed gluten-free products and that quality of life on the diet is measurably impaired, particularly in its social dimensions. Every patient with CeD, NRCD, or RCD requires a referral to a registered dietitian experienced in CeD management, not only at diagnosis but at regular follow-up intervals for nutrition management. More research is needed to evaluate the necessary precautions to take in preventing gluten cross-contact to improve disease outcomes and food-related quality of life in individuals with CeD.

## 5. Inflammatory Bowel Diseases

The pathogenesis of IBD has been hypothesized to stem from interactions among genetic and environmental factors. A systematic review and meta-analysis of nine studies with 54,580 individuals revealed the consumption of a Western diet pattern to be associated with an increased risk of IBD [68]. Similarly, in the prospective Nurses’ Health Study and Health Professionals Follow-up Study with 245,112 individuals representing 5,468,444 person-years of follow-up, the increased consumption of ultra-processed foods commonly found in a Western diet was associated with an increased risk of Crohn’s disease (CD) [69]. By contrast, in an analysis of 83,147 adults who participated in the prospective Cohort of Swedish Men and Swedish Mammography Study, the consumption of the Mediterranean diet was associated with a lower risk of CD [70]. These trends of diet and IBD risk are further reflected in regional variations of IBD incidence [71]. From a mechanistic standpoint, diets can exert a significant effect on intestinal inflammation. Putative mechanisms include the reduced exposure to pro-inflammatory food antigens, the promotion of favorable microbiome and metabolomic profiles, the maintenance of the gut mucosal barrier, and the modulation of gut immunity [72].

### 5.1. Dietary Therapies for Inflammatory Bowel Disease

Given patients’ significant interest in dietary therapy and more studies demonstrating their potential efficacy, there has been a recent spike in the number of prospective controlled trials. Over the past five years, there have been over 40 such trials presented at conferences or published, compared with less than 20 in the preceding 5 years and less than 5–10 in the preceding 5-year blocks.

Despite the correlation of refined carbohydrates, trans-unsaturated fats, and ultra-processed food intake with the pathogenesis of IBD, the isolated avoidance of these foods has not yet been clearly linked to the control of disease activity in individuals with IBD [73,74,75,76]. Nonetheless, the combined exclusion of these pro-inflammatory foods—and the encouraged increase in anti-inflammatory foods (e.g., fruits and vegetables)—constitutes a key premise of anti-inflammatory diets, accompanied by supporting research evidence of benefit.

#### 5.1.1. Enteral Nutrition

EEN has been demonstrated to be effective for CD, where several medical and nutrition societies now recommend it as a first-line corticosteroid-sparing therapy for the induction of remission in pediatric CD [77]. The type of formula (i.e., elemental, semi-elemental, and polymeric) has otherwise not been demonstrated to differ in efficacy [78]. As EEN can be challenging to follow, particularly over an extended period, there has been significant interest in exploring other forms of anti-inflammatory nutrition therapy. Partial enteral nutrition (PEN) combines the potential benefits of enteral nutrition (EN) formulae with a solid food diet, making it a more palatable treatment alternative. In an early study published in 1983, 28 malnourished patients with CD were randomized to either receiving supplemental EN with a regular diet at baseline or after two months [79]. The study was primarily designed to assess the improvement in malnutrition, although the secondary endpoints included some metrics of inflammation. Supplemental EN was noted to lead to a reduction in serum orosomucoid levels (a historically used marker of inflammation), but there was no change in disease activity scores or the erythrocyte sedimentation rate (ESR). In more recent years, there have been over twenty controlled trials of PEN for CD using different combinations of solid food diets. The landmark trial that reinvigorated interest in the use of PEN for the treatment of CD was a multi-national trial by Levine et al. [80]. In this RCT, 74 children with mild-to-moderate CD were randomly assigned to either receive EEN or PEN (50% of calories from EN and 50% of calories from the Crohn’s Disease Exclusion Diet [CDED]) for 6 weeks. PEN was found to have an equivalent efficacy with EEN in achieving a clinical response and remission, although PEN had a significantly greater tolerance (97.5% vs. 73.7%, *p* = 0.002).

The data on the use of EEN or PEN for ulcerative colitis (UC), on the other hand, are more sparse and less suggestive of a benefit. An open-label RCT compared PEN with 75% of calories from Mediterranean and low FODMAP diets versus dietary counseling for healthy eating [81]. The disease activity scores decreased similarly in both intervention arms, although CRP significantly declined in the PEN arm but not the control arm. An unpublished observational study in 60 patients with mild-to-moderate UC found PEN (EN combined with an exclusion diet) to be significantly associated with the absence of rectal bleeding in week 4, when compared with a habitual diet [82]. There was otherwise no significant difference in clinical remission or need for corticosteroids between intervention arms.

#### 5.1.2. Crohn’s Disease Exclusion Diet

Besides spurring interest in PEN, the RCT by Levine et al. also showcased the potential benefit of CDED. The CDED largely excludes foods thought to be pro-inflammatory (e.g., red meat, wheat, dairy, emulsifiers, maltodextrin, and carrageenan), while encouraging the increased consumption of fruits and vegetables. A key feature of the diet is the daily intake of mandatory foods—two cooked and cooled potatoes, two bananas, and one apple—for 12 weeks, providing resistant starch intended to support the microbiome diversity and composition. After the initial 6-week phase of the trial, participants originally assigned to the PEN arm transitioned to receiving 75% of their calories from CDED and 25% of their calories from EN [80]. Those who were originally assigned to the EEN arm transitioned to receiving 75% of their calories from their habitual diet. In week 12, those who continued with the CDED had a greater sustained clinical remission (75.6% vs. 45.1%, *p* = 0.01) and normal CRP (75.9% vs. 47.6%, *p* = 0.04). In a separate open-label RCT by Pasta et al. involving 21 adults with CD assigned to CDED and 21 to a Mediterranean diet, the CDED led to a significantly greater proportion of individuals achieving clinical remission in weeks 12 and 24 [83]. There was a significant decrease in ESR for the CDED group in week 12 and, otherwise, no difference within and between groups for ESR, CRP, and fecal calprotectin.

#### 5.1.3. Mediterranean Diet

The Mediterranean diet has long been considered a healthy plant-based dietary pattern, characterized by a high intake of dietary fiber and monounsaturated fats, and is associated with benefits to cardiovascular health and the gut microbiome [84]. There has similarly been interest in its use as an anti-inflammatory diet. In an RCT of 50 adolescent patients with mild-to-moderate IBD, when compared with a habitual diet, the Mediterranean diet led to significantly lower CD and UC activity indices, CRP, and calprotectin in week 12 [85]. In an RCT of the Mediterranean diet versus standard dietary counseling in 20 adults with mildly-to-moderately active CD at baseline, the Mediterranean diet led to greater clinical remission in week 8 (80% vs. 40%) [86]. In the overall cohort of 78 participants, fecal calprotectin significantly decreased in the Mediterranean diet group but not in the control group. When compared with other anti-inflammatory diets, the aforementioned RCT by Pasta et al. found CDED to be superior to the Mediterranean diet for the achievement of clinical remission in weeks 12 and 24 [83]. In a large RCT with 197 adults with mild-to-moderate CD, we found the Mediterranean diet to be similar to the Specific Carbohydrate Diet (SCD) for achieving symptomatic remission, calprotectin response, and CRP response [87]. On the other hand, an RCT of the Mediterranean diet and SCD in adults with mild-to-moderate UC was terminated early at 17 participants due to the lack of an observed benefit (change in clinical remission: 11.1% for Mediterranean diet vs. 0% for SCD, *p* = 0.998), a worsening of the disease, and the high dropout rate [88].

#### 5.1.4. Specific Carbohydrate Diet

The SCD, which restricts carbohydrates in the form of disaccharides and polysaccharides along with grains, most processed foods, and certain dairy products, was initially conceived for celiac disease in the 1920s but was more recently popularized in the 1980s by Elaine Gottschall after finding it to have benefitted her daughter with UC [89]. Despite the vigorous interest and application of this diet for IBD, there are few rigorous studies on it. The RCT by Lewis et al. found the SCD to be similar to the Mediterranean diet in CD [87]. The RCT comparing the SCD with the Mediterranean diet for UC was terminated early for logistical and efficacy reasons [88]. An RCT that compared the SCD with a whole-food diet for CD found a 100% clinical remission and no difference between diets in week 12, although the analyzed sample size was very small (n = 8 vs. 2) [90].

#### 5.1.5. Low FODMAP Diet

The low- FODMAP diet has been effective in IBS (see Section 2) and has thus been studied for individuals manifesting an IBD–IBS overlap [91]. In an RCT with 52 patients with quiescent IBD, participants were randomized to a low- FODMAP diet or control diet [92]. The change in IBS Severity Scoring System (IBS-SSS) scores did not significantly differ between diet arms after 4 weeks (−67 vs. −34; *p* = 0.075), but was significantly different in the subgroup with UC (−77 vs. −29; *p* = 0.031). There were nonetheless significant differences between the diets for the 50% reduction in IBS–SSS (33% vs. 4%; *p* = 0.012), adequate relief (52% vs. 16%; *p* = 0.007), and stool frequency (1.7 vs. 2.1; *p* = 0.012). In a larger RCT with 89 patients with quiescent or mild-to-moderate IBD and concurrent IBS-like symptoms (based on Rome III criteria), participants were randomized to a low-FODMAP diet or habitual diet for 6 weeks [93]. Those assigned to the low- FODMAP arm had significantly lower IBD-SSS scores and higher increases in quality-of-life scores. Besides the treatment of IBS-like symptoms, the low- FODMAP diet has been investigated for the treatment of inflammation. In an RCT of 60 patients with quiescent or mild-to-moderate IBD, the low- FODMAP diet, but not the control diet, led to a decrease in the Harvey–Bradshaw Index and fecal calprotectin [94].

#### 5.1.6. Natural Whole-Food Diet

As multiple diets have thus far been found to be effective for the induction and maintenance of remission in IBD, we hypothesized that diet quality drives the anti-inflammatory benefit. In an RCT with 28 individuals with mildly-to-moderately active CD, participants were assigned to either a natural whole-food diet or the continuation of their habitual diet [95]. Stratified by the diet quality using the Healthy Eating Index score, those with a higher diet quality had a significantly higher proportion of individuals achieving clinical remission. A higher diet quality was also associated with a significantly greater reduction in fecal calprotectin. An RCT in 83 pediatric patients with mild-to-moderate CD found a whole-food diet to be comparable to EEN for the induction of clinical remission (per-protocol: 66% vs. 76%; *p* = 0.92) and normalization of fecal calprotectin (38% vs. 41%; *p* = 0.79) and CRP (49% vs. 65%; *p* = 0.53) [96].

#### 5.1.7. Miscellaneous Diets

Several other diets have been considered for IBD, but the data are less robust or less supportive. Emerging controlled studies have shown the potential benefit of several dietary variants, such as the semi-vegetarian diet or low refined carbohydrate diet [97,98]. Thus far, there has been no clear benefit of diets reducing or eliminating cow’s milk protein, gluten, carrageenan, microparticles, red meat, or emulsifiers for the induction of remission in IBD [98,99]. Single-arm diet studies have also been conducted, but they are not discussed as it would be difficult to differentiate between dietary effects and the natural course of disease.

### 5.2. Summary

Given the current state of the evidence, there has been consistent support across the epidemiologic, observational cohort, and intervention studies to indicate that diet influences IBD pathogenesis and disease activity. For the induction of remission, EEN has been established to be a corticosteroid-sparing treatment. However, consumption can be challenging. Fortunately, emerging studies have shown PEN to similarly be effective, thus opening a broader menu of dietary options (Table 4). Controlled studies also suggest the potential benefit to varying degrees of solid food diets, such as the CDED, Mediterranean diet, semi-vegetarian diet, and natural whole-food diet for the induction of clinical remission and reduction in inflammation. Common themes across these purportedly beneficial diets are combined with the avoidance of pro-inflammatory foods (e.g., refined carbohydrates, saturated fats, emulsifiers, and ultra-processed foods), and the consumption of anti-inflammatory foods (e.g., fruits and vegetables) is encouraged. The isolated reduction in refined carbohydrates, such as with the low FODMAP diet, has also shown promise, although most of the studies have so far focused on IBS-like symptoms among patients with quiescent IBD. Most of the studies have focused on CD. Nonetheless, these demonstrated effects of diet highlight its importance as part of the conversation with patients in clinical practice. Notably, appropriate diet counseling would be important to ensure an adequate adherence to the overall diet and the exclusion of food incriminated in IBD pathogenesis.

## 6. Gastroparesis

Gastroparesis is a chronic motility disorder characterized by delayed gastric emptying in the absence of mechanical obstruction. The causes of gastroparesis are broadly classified as diabetic, post-surgical or iatrogenic, idiopathic, or neurologic [100]. In all cases of gastroparesis, disruptions to the autonomic and enteric nervous system, vagus nerve, and smooth muscle cells, including the interstitial cells of Cajal, pacemaker cells, and network of Plexi, result in sensory dysfunction and impaired motor activity. Symptoms of gastroparesis include nausea, abdominal pain, vomiting, early satiety, or postprandial fullness [101]. Gastroparesis can result in significant nutritional challenges and impairments to quality of life. Inadequate nutrition intake is common, with an estimated 64% of individuals not meeting energy requirements [102]. For those unable to meet nutritional needs by mouth, nutrition support via tube feeding or PN may be indicated, posing additional risks [103]. Importantly, due to symptom association with oral intake, disordered eating risk is increased in gastroparesis, with a survey indicating 77% of adults with gastroparesis screened positive for avoidant restrictive food intake disorder (ARFID) [104].

### 6.1. Dietary Therapies for Gastroparesis

The current state of clinical practice using diet for gastroparesis is more based on physiological principles than clinical studies. Patients with gastroparesis are commonly advised to follow a diet low in fat and dietary fiber, replace solids with liquids, and consume smaller, more frequent meals. In surveys of patients with gastroparesis, “figuring out what to eat” and “managing symptoms” ranked highest in aspects of living with gastroparesis that caused the most difficulty [105].

Foods that are often poorly tolerated in gastroparesis include fatty, acidic, spicy, and roughage-based foods. A 2015 survey on food toleration and aversion in 45 individuals with idiopathic or diabetic gastroparesis found some of the most aggravating foods to include orange juice, fried chicken, cabbage, sausage, pizza, peppers, onions, tomato juice, lettuce, and coffee [102]. Conversely, tolerated foods were higher in carbohydrate content, bland, sweet, salty, and starchy, and included foods such as saltine crackers and gelatin. This study affirms current recommendations to modify dietary fiber and fats and suggests that common gastrointestinal irritants may worsen symptoms.

Current dietary practices recommend modifying fiber in gastroparesis; however, fiber characteristics (i.e., viscosity, solubility, and particle size) can influence tolerability. A 2008 study by Olausson et al. evaluated small- versus large-particle fiber in seven diabetic patients with gastroparesis versus seven healthy controls [106]. Participants received two test meals: a large-particle meal consisting of 100 g roast beef slices, 150 g grated raw carrot, 40 g pasta, and 5 g canola oil versus a matched-portion small-particle meal consisting of minced/baked beef, boiled carrots mixed in a food processor with pasta, and canola oil. Postprandial gastric emptying indicated a prolonged lag phase in the large-particle meal, at 15.1 min in the large-particle meal versus 1.4 min in the small-particle meal, highlighting the role of particle size in tolerance. Similarly, a 2014 RCT of 56 patients with diabetic gastroparesis evaluated the particle size among a small-particle diet versus control diet, both consisting of three meals and three snacks daily over 20 weeks (see Table 5 for the further characterization of particle sizes) [107]. In the intervention diet, all gastrointestinal symptoms (nausea, vomiting, early satiety, fullness, bloating, regurgitation, and heartburn), except abdominal pain, improved significantly. Notably, the intervention diet group consumed more fat than the control diet group, suggesting multiple dietary components may influence symptoms.

To assess fiber viscosity, a 2021 pilot clinical study of 10 female participants with mild to moderate gastroparesis evaluated fiber supplements (10 g) including partially hydrolyzed guar gum, gum Arabic, and psyllium husk for their tolerability and impact on short-term blood glucose regulation. Both partially hydrolyzed guar gum and gum Arabic were better tolerated for postprandial fullness, early satiety, and bloating/distension compared with psyllium husk [108]. This study indicated a role for low-viscosity fibers in gastroparesis diet modification versus outright fiber avoidance.

Dietary fat reduction is commonly recommended in gastroparesis, as fats often worsen symptoms and delay gastric emptying via increased cholecystokinin. A 2015 study by Homko et al. evaluated dietary fat tolerance in gastroparesis across four meal types: high-fat solid meal, high-fat liquid, low-fat liquid, and low-fat solid [109]. The meals were matched in their nutritional composition with 260 kcals and 13 g dietary fat for the high-fat arm and 1.5–2 g fat for the low-fat arm. Participants noted the highest symptom scores in the high-fat solid meal compared with the other test meals, with more than double the nausea symptom scores, increased bloating, upper abdominal pain, stomach fullness, and heartburn, and there were no significant differences in total symptom score between the low-fat solid, high-fat liquid, and low-fat liquid diets.

### 6.2. Summary

While clinical data are lacking to guide dietary therapies in gastroparesis, current best practices include modifying fiber based on form and particle size, consuming smaller, more frequent meals, reducing dietary fat or modifying form of fat (e.g., liquid), replacing solid foods with liquids where indicated, and sitting upright or engaging in gentle movement after mealtime. Further studies are needed to better characterize the global patterns of dietary needs in gastroparesis, as well as to inform strategies to further individualize diet based on etiology, severity, and symptom presentation.

## 7. Short Bowel Syndrome and Intestinal Failure

SBS is a form of intestinal failure where impairments in intestinal digestion, absorption, and motility result in the malabsorption of an oral diet. Symptoms include chronic diarrhea, dehydration, and nutrient deficiencies. The European Society for Clinical Nutrition and Metabolism (ESPEN) defines intestinal failure as a “reduction of gut function below the minimum necessary for the absorption of macronutrients and/or water and electrolytes, such that intravenous supplementation is required to maintain health and/or growth” [110]. When a reduction in absorption does not require intravenous fluid or nutritional support, the reduction is referred to as intestinal insufficiency or intestinal deficiency, where patients with SBS may have symptoms, but are able to maintain weight and other nutritional parameters without nutrition support.

Intestinal adaptation describes the ability of the remaining small bowel to enhance its absorptive capacity for oral and/or enteral nutrients and fluids [111]. In SBS, the site and extent of resection determine the physiological consequences. After jejunal resection, the ileum often adapts well, but the loss of enteric hormonal regulation may trigger gastric hypersecretion, increasing acid delivery to the small bowel and thus increasing diarrhea and electrolyte losses. Ileal resection is more challenging, as the jejunum has a limited ability to compensate for the loss of the ileal function.

Diet and pharmacological interventions to slow transit are essential. The entry of oral or enteral nutrients into the small bowel stimulates adaptive processes, including crypt cell hyperplasia, villus growth, and increased enteric hormone synthesis, which gradually slows intestinal transit and prolong nutrient contact within the small bowel, thereby enhancing digestive and absorptive function. The enteric continuity of a colon and ileocecal valve also contributes an additional five feet of bowel to support a gradual increase in nutrient absorption. This whole adaptive process evolves over a period of two years or longer.

### 7.1. Nutrition Support for Short Bowel Syndrome

#### 7.1.1. Enteral Nutrition

For SBS, EN should be initiated after surgery as soon as the patient is stable to stimulate intestinal adaptation, via a gastrostomy tube to maximize proximal nutrient delivery and interaction with the small bowel. Continuous low-rate EN promotes adaptation by increasing intestinal blood flow, stimulating pancreatic and biliary secretions, and activating intestinal hormones and neural pathways [111]. Most of the evidence guiding clinical practice for EN in SBS comes from small studies. An earlier, small, randomized, prospective crossover study of 15 adults with SBS evaluated the impact of EN on nutrient absorption [112]. Participants underwent seven days of exclusive 24 h EN, followed by seven days of an unrestricted oral diet; a subset also completed a phase combining oral intake with 1000 kcal/day of supplemental EN, separated by washout periods of one to four weeks. The net absorption of protein, fat, and total energy was significantly higher with exclusive tube feeding: 72% vs. 57% for protein, 69% vs. 41% for fat, and 82% vs. 61% for total energy. Supplemental EN also improved absorption compared with oral intake alone. These result in both exclusive and supplemental EN enhancing nutrient absorption in post-operative SBS.

Polymeric formulae are generally preferred, because their intact nutrients stimulate digestion and absorption processes [110]. Elemental or semi-elemental formulae are used when patients have an intolerance to polymeric feeds (e.g., increased diarrhea). Bolus feeds may be poorly tolerated due to larger volumes, whereas continuous pump feeds deliver nutrients gradually at a slower rate. If weight loss or further symptoms are seen with increasing EN volume, the rate should be reduced to the last well-tolerated level before further progression. EN discontinuation is dependent on a patient’s adequacy and tolerance of an oral diet and the maintenance of nutritional parameters such as weight and hydration.

#### 7.1.2. Parenteral Nutrition

PN is initiated in the post-operative state once the patient is stable for initiation and serves as the primary source of nutrition while tolerance to an oral diet and EN is established. PN weaning to partial or complete independence depends on several key factors, including having more than 75 cm of small bowel with an intact colon or ileocecal valve, the absence of disease in the remnant intestine, and evidence of intestinal adaptation. Long-term outcomes depend on adherence to the diet, hydration, and antidiarrheal interventions [113,114]. A PN wean is usually initiated by continuing with daily PN while reducing its calories by 10–30%, often starting with a 300–500 calorie reduction. If the intake from the diet remains stable without weight loss, dehydration, and other symptoms, PN can be further weaned either with a daily reduction in calories or through PN “holidays” for days off the regimen. PN can be stopped once the patient’s average daily PN intake is between 500–700 kcal and an adequate intake is achieved through diet. IV fluids may be required to maintain hydration during this process [113,114].

Achieving a PN wean and/or independence is essential in order to reduce the risk of PN-associated liver disease (PNALD). PNALD is identified when hepatic transaminases rise to ≥1.5 times the upper limit of normal, which can occur even within 1–3 weeks of PN initiation [115]. Risk factors include reduced enteric stimulation, leading to gallbladder stasis and bacterial translocation, as well as continuous PN or high dextrose/lipid loads, which promote hyperinsulinemia and lipogenesis. Strategies to reduce PNALD focus on maintaining enteral stimulation, using cyclic PN over 10–16 h, and incorporating PN “holidays” when feasible. Dextrose should be limited to 15–20 kcal/kg/day and lipids to 0.5–1 g/kg/day to minimize lipogenesis. Omega-3–lipid emulsions support anti-inflammatory eicosanoid production and hepatic fatty acid beta-oxidation.

### 7.2. Dietary Therapies for Short Bowel Syndrome

Diet should commence after surgery as medically feasible. Earlier studies in SBS demonstrated that nutrient absorption varies widely based on remnant bowel anatomy, particularly the presence or absence of an ileum and colon, which continues to guide contemporary dietary therapy. Diet is thus tailored based on anatomic considerations, as well as tolerance, access, lifestyle, culture, and food preferences.

#### 7.2.1. Calories

Adaptive hyperphagia describes the physiological increase in oral intake that compensates for reduced absorption. A retrospective study of 59 adults with SBS evaluated hyperphagia, defined as a Food Intake Ratio (FIR) exceeding 1.5 times resting energy expenditure (REE) [116]. FIR was calculated as the highest recorded oral caloric intake divided by the REE. Hyperphagia was observed in 83% of participants and was independent of sex, PN use, and remaining bowel length, although a higher body mass index was associated with a lower FIR. Patients with a jejunocolonic (n = 31) or jejunoileal anastomosis (n = 9) had significantly higher FIRs than those with an end-jejunostomy (n = 18) (*p* < 0.05). Patients with colonic continuity had higher FIRs, while the remaining small bowel length had no significant effect on intake. The results suggest that the colonic presence facilitates a greater oral intake in SBS.

#### 7.2.2. Carbohydrates

Carbohydrate intake has variable effects depending on its composition, type, and degree of fermentability. In a study of 14 adults, a high-carbohydrate, low-fat diet reduced stool energy losses by approximately 480 kcal/day in patients with a colon, whereas no benefit was observed in those with a jejunostomy [117]. In another study of 17 adults with SBS, the ingestion of 20 g of lactose was well-tolerated, with both milk and yogurt consumed without an increase in output, supporting symptom-guided trials rather than routine dairy and lactose elimination [118]. Additionally, a 4-day crossover study in 12 adults with non-SBS ileostomies demonstrated that a high FODMAP diet increased the effluent volume by 22%, primarily due to fructans and sorbitol, highlighting the potential impact of poorly absorbed fermentable carbohydrates on the output as well [119]. The intake of carbohydrates as complex starches and dietary fiber is encouraged in individuals with a remaining colon to support colonic bacterial fermentation and the production of short-chain fatty acids. By contrast, individuals without a colon often require a personalized carbohydrate intake based on tolerance, as carbohydrates may increase the osmotic load and worsen fluid and electrolyte losses. Fiber in a small particle size may be better tolerated. Concentrated sweets should be limited as these foods may have an osmotic effect on the remaining small bowel and contribute to diarrhea.

#### 7.2.3. Protein

In SBS, protein digestion and amino acid absorption are usually preserved. The ESPEN guidelines recommend an increased protein intake (approximately 1.0–2.0 g/kg/day) in patients with SBS to maintain a positive nitrogen balance [120]. Protein in the form of whole foods are preferred, as they supply not only essential amino acids but also additional nutrients that support the overall nutritional status. Animal proteins are of high biological value as they provide all essential amino acids. Plant proteins also provide fiber, and their use should be tailored to the individual’s tolerance to fiber.

#### 7.2.4. Fat

Fat and bile acid absorption depends on the remaining small bowel anatomy. The interruption of enterohepatic circulation following an ileal resection of greater than 100 cm that has colonic continuity prevents bile salts from being recycled from the ileum to the liver. Consequently, bile salts pass into the colon, contributing to bile-salt diarrhea. In contrast, in patients with a jejunostomy or ileostomy, dietary fat does not appear to significantly increase the effluent [121]. An earlier study in patients with end-jejunostomy showed that a higher fat intake (60% of total calories) increased steatorrhea and divalent cation losses (calcium, magnesium, zinc, and copper) without consistently affecting the volume of the ostomy output or the loss of sodium and potassium [122].

For patients with an intact colon, a low-fat diet is recommended, but very strict fat restriction is not needed, as it can lead to a significant reduction in caloric intake. Both animal-based fats (e.g., meat, poultry, and fish) and plant-based fats (e.g., avocado, nut butters, and olive oil) can be consumed in tolerated amounts to provide sources of long-chain triglycerides for essential fatty acids. Small case reports and series indicate that supplementation with medium-chain triglycerides may help increase the production of short-chain fatty acids [123]. For patients with an ostomy, dietary fat can typically be continued without restriction, with monitoring for steatorrhea or micronutrient losses in those with high-output states.

#### 7.2.5. Oxalates

Under normal physiology, oxalate binds to calcium in the gut and is excreted in the stool. In SBS, fat malabsorption alters this process: unabsorbed fat binds to calcium, leaving oxalate free to pass from the small intestine into the colon, where it can be reabsorbed and ultimately reach the kidneys. Dehydration may impair the renal excretion of oxalate, leading to its accumulation and increasing the risk of kidney stones. Kidney stones are common in adults with SBS (18%) with risk factors including jejunoileal anastomosis with colon-in-continuity, a longer duration of SBS, and higher levels of serum creatinine [124]. In an earlier study of 84 adults with less than 200 cm of jejunum, nephrolithiasis occurred solely in those with a functional intact colon. Over a long-term follow-up at 5 years, 24% of patients with a colon developed symptomatic nephrolithiasis, all of which were calcium oxalate stones, whereas no cases were observed in patients without a colon in continuity [125]. In patients with SBS who have an intact colon, a low oxalate diet is often recommended when there is a history of nephrolithiasis and/or low urine output ≤ 1200 mL/day. Adequate hydration between the oral diet and/or intravenous fluids is also essential [126].

#### 7.2.6. Hydration

Dehydration can arise as excess thirst, dry mouth, rapid weight loss, fatigue, and hypotension, and its long-term consequences include fatigue, kidney injury, and kidney disease. The loss of bowel surface in SBS reduces sodium and water absorption, increasing the risk for dehydration. The colon, if intact, reabsorbs sodium and water to further optimize the electrolyte and fluid balance [127]. Patients with stomas who lose 1500 mL or more of effluent within 24 h are also considered at a high risk for dehydration. Hypomagnesemia can also arise with sodium and water losses. Oral rehydration solutions (ORS) optimize hydration by utilizing the sodium–glucose co-transport system, in which sodium and glucose are absorbed together, creating an osmotic gradient that supports passive water absorption [128]. The optimal ORS contains 20–40 g of sugar (5–10 teaspoons) and 90–120 mEq of sodium per liter, with 1 teaspoon of salt providing 104 mEq of sodium. An initial ORS volume of 500 mL/day can be considered and gradually increased through diet or through a gastrostomy tube to meet fluid needs. Hypertonic fluids should be consumed in lower amounts, such as fruit juices, soda, or sweetened sports drinks, as they draw water into the gut lumen, increasing the risk for diarrhea and dehydration—hypotonic fluids as well, including plain water or tea, as these fluids can dilute electrolytes and contribute to further losses. As tolerance can vary, the fluid type, amount, and timing should be personalized per needs and preferences. Intravenous fluids may be required to maintain hydration as needed.

### 7.3. Summary

SBS is a form of intestinal failure where a reduced small bowel length leads to the impaired absorption of nutrients, fluids, and electrolytes, thereby causing diarrhea, dehydration, and nutrient deficiencies. Management relies on intestinal adaptation and a combination of dietary modifications, EN to enhance intestinal adaptation, and PN to deliver intravenous nutrients to optimize the nutritional status and hydration (Table 6). Over time, the remaining small bowel may improve its absorptive capacity, allowing some patients to reduce or discontinue intravenous support.

Diet therapy is individualized based on bowel anatomy and tolerance, focusing on optimizing the energy intake and adjusting the nutrient intake to enhance absorption and reduce stool or stoma output.

## 8. Micronutrient Deficiencies

Micronutrient deficiencies are common across gastrointestinal disorders. Inflammation, altered anatomy, and restrictive intake frequently contribute to micronutrient deficiencies. Routine nutritional screening and laboratory monitoring are essential. Because inflammation can alter acute phase reactants and complicate interpretation, micronutrient testing is best performed in an outpatient setting.

In CeD, malabsorption contributes to micronutrient deficiencies due to gluten-induced damage to the small bowel. Iron deficiency is the most reported nutritional deficiency in CeD, affecting 14% to 41% of adult patients even on a GFD with biopsy-proven mucosal remission [129]. Vitamin D deficiency is also very common, occurring in up to 59% of individuals with CeD [57]. B-group vitamin deficiencies, particularly B12, folate, and B6, remain significant on the GFD, because many commercially available gluten-free products are made with refined starches and flours that lack the nutrient density of their wheat-based counterparts and are not required to be enriched or fortified [130]. Zinc, magnesium, calcium, and fat-soluble vitamins A and E are also micronutrients to be assessed [57]. Due to the high prevalence of micronutrient deficiencies both at the diagnosis of CeD and on a long-term GFD, clinicians should track the trends in folate, ferritin, transferrin saturation, and 25-hydroxyvitamin D levels at regular [57].

In gastroparesis, a patient survey indicated deficiencies in vitamin D, vitamin E, vitamin K, folate, calcium, iron, magnesium, and potassium [103]. Other common nutrients of concern in gastroparesis include vitamin B6, vitamin C, and zinc [131]. It is recommended that all individuals with gastroparesis consume a daily multivitamin, and yearly labs should include a metabolic panel with liver tests, vitamin B12, folate, vitamin D, and an iron panel, and, in certain instances, fat-soluble vitamins and minerals including zinc, copper [132].

In SBS, malabsorption also contributes to common micronutrient deficiencies, which include fat-soluble vitamins (vitamin A, D, and E) and vitamin B12 deficiency following an ileal resection greater than 60 cm, and minerals such as copper, zinc, and iron. A recent retrospective study evaluated micronutrient alterations in adults with SBS after being off PN for at least a year [133]. Of 40 patients, 64% had SBS subtype II, and 75% were mainly on vitamin D or B12 supplements. Despite this, 79% had at least one or more fat-soluble vitamin deficiency (most commonly vitamin D) and 83% had at least one or more trace element deficiency (selenium, copper, or zinc). The results show the need for ongoing micronutrient monitoring after PN weaning. The American Gastroenterological Association (AGA) advises that all patients with SBS—regardless of PN use—should have a baseline and annual evaluation of micronutrients [134].

## 9. Discussion and Future Perspectives

Emerging evidence highlights the shared mechanisms (e.g., an aberrant immune response, the disruption of gut mucosa, and gut microbial dysbiosis) across several gastrointestinal disorders that contribute to their pathogenesis. EoE, CeD, and IBD are primarily immune-mediated disorders, while IBS and gastroparesis have an underlying component of immune activation. Inflammation can disrupt the gut epithelial barrier, increase gut permeability, permit microbial translocation that activates submucosal immune cells, and propagate the inflammatory response [135]. Gut microbial dysbiosis has been strongly associated with disease pathogenesis, such as with IBS, IBD, and CeD. Their putative effects may be direct (e.g., interaction with immune cells) or indirect (e.g., the generation of immunomodulating metabolites) [136]. However, the role of the gut microbiota as a causative factor or effect remains unclear [137,138]. The further involvement of neuro-myoenteric interactions and dysmotility are hallmarks of disorders such as DGBI, gastroparesis, and SBS.

Among the disorders discussed in this review, food plays a major role in their pathophysiology and disease presentation. Food as an antigen can directly and indirectly lead to immune activation. Alternatively, malabsorption stemming from the disruption of the gut mucosa (e.g., with EoE, CeD, and IBD), altered motility (e.g., with IBS, gastroparesis, and SBS) or reduction in absorptive surface area (e.g., SBS) can lead to diarrhea and nutrient deficiencies. In these instances, dietary therapies are designed to address the underlying pathogenic factors and/or mitigate food-related effects of active disease. As such, diet is a crucial component of a multi-pronged strategy for managing gastrointestinal disorders (Table 7).

The effectiveness of dietary therapy is nonetheless strongly dependent on dietary adherence. Psychosocial and cultural factors have strong influences on dietary adherence and symptom perception, which will require the individualization of dietary therapies to reduce challenges and improve implementation. Food culture shapes food choices and the willingness to adopt new ways of eating [139]. Patients also often identify certain foods as triggers and may severely restrict or eliminate them to reduce symptoms. If continued, they may also contribute to an altered nutritional status with weight loss, micronutrient deficiencies, and disordered eating such as avoidant-restrictive food intake disorder [140,141]. Culturally responsive nutrition care, alongside psychological support, is needed to reduce unnecessary food restriction and optimize dietary intake for symptom management.

This review summarizes the current practice and recommendations for dietary therapy based on available research. Admittedly, the overall certainty of evidence remains low or very low for many interventions, and nutrition research continues to lag behind pharmacologic studies, highlighting the need for additional high-quality studies. Future directions to improve the effectiveness of diet therapies could include investigating diet, behavioral, and cultural strategies to optimize diet adherence and advancing precision-nutrition interventions with the aid of artificial intelligence that match diets to individual genetics, clinical phenotypes, biochemical markers, and environmental exposures. Further investigation into beneficial microbial taxa or, more importantly, their functional metabolomic profiles would help in the development of microbiome-guided dietary therapies. Nonetheless, despite these gaps in evidence, “food as medicine” can still be applied today in clinical practice as it has been over the past millennia.

## Figures and Tables

**Table 1:** Current evidence on specific dietary therapies for disorders of gut–brain interaction.

Diet	Irritable Bowel Syndrome	Chronic Constipation	Functional Dyspepsia
Fiber supplementation	Some benefit of soluble—but not insoluble—fiber for global IBS symptoms demonstrated in systematic review and meta-analysis of 14 RCTs with 906 participants [19].	Some benefit of fiber for constipation demonstrated in systematic review and meta-analysis of 16 RCTs with 1251 participants [20].	Insufficient data.
Low FODMAP diet	Some benefit for improvement of global IBS symptoms, abdominal pain, and bloating, but not bowel habits in systematic review and meta-analysis of 28 RCTs with 2338 participants [23].	Insufficient data.	Single-blind RCT with 105 participants found no difference between low FODMAP diet and traditional dietary advice in overall symptoms improvement. Subgroup analysis found benefit of low FODMAP diet for those with subtypes of postprandial distress syndrome or bloating [32].
Mediterranean diet	Some benefit for improvement of overall symptoms.	Insufficient data.	Insufficient data.
IgG-based elimination diet	Some benefit for reduction of abdominal pain in RCT with 223 participants [24].	Insufficient data.	Insufficient data.
Smaller, more frequent meals with low-fat content and avoiding food triggers	Insufficient data.	Insufficient data.	General recommendation but not assessed in RCTs.

Abbreviations: FODMAP, fermentable oligo-, di-, monosaccharides, and polyols; IBS, irritable bowel syndrome; RCT, randomized controlled trial.

**Table 2:** Current evidence on specific dietary therapies for eosinophilic esophagitis.

Diet	Eosinophilic Esophagitis
Exclusive enteral nutrition	Reduction in eosinophils in 10 children with GERD and having had a Nissen fundoplication [39]. Reduction in dysphagia, vomiting, abdominal pain, and eosinophils in 346 participants with chronic GERD and eosinophilic esophagitis [40]. Reduction in eosinophils in 18 adults with eosinophilic esophagitis [41].
1-, 2-, 4-, and 6-food elimination diets	Some benefit for eosinophilic esophagitis.

Abbreviations: GERD, gastroesophageal reflux disease.

**Table 3:** Role of dietary therapies for celiac disease.

Education on the gluten-free diet
Dietary sources of gluten; Read ingredient labels of packaged foods, and dietary supplements; Incorporate naturally gluten-free grains; Precautions to prevent gluten cross-contact in food preparation; Questions to ask when dining out and traveling.
Management of malnutrition and nutrient deficiencies
Common deficiencies at diagnosis: iron, zinc, vitamin D, vitamin A, and vitamin E; Nutrients low in gluten-free processed foods: ○ B vitamins: B1, B2, B6, folate, and B12; ○ Iron; ○ Dietary fiber; Weight regain including muscle.
Assess gluten-free diet adherence
Frequency of intentional gluten intake; Sources of unintentional gluten intake: ○ label reading; ○ cross-contact in home kitchen; ○ cross-contact when dining out; ○ frequency and quantity of oats consumed; Barriers to dietary adherence.
Manage ongoing symptoms
Adequate dietary fiber; Low FODMAP diet; Nutrition therapy for co-occurring disorders (SIBO, microscopic colitis, etc.).
Nutrition support for malabsorption in refractory celiac disease
Oral vs. intramuscular/intravenous micronutrient repletion; Oral nutrition shakes to mitigate weight loss, and protein loss; Manage protein-losing enteropathy; Enteral nutrition: peptide or elemental formula considerations; Parenteral nutrition: in cases of intestinal failure.
Screen for disordered eating
Intentional gluten intake to prevent weight gain; Binge eating and purging behaviors; Discomfort with body composition changes; Hypervigilance to gluten cross-contact; Impaired quality of life and social engagement.

Abbreviations: FODMAP, fermented, oligo-, di-, monosaccharides, and polyols; SIBO, small intestinal bacterial overgrowth.

**Table 4:** Current evidence on specific dietary therapies for inflammatory bowel disease.

Diet	Crohn’s Disease	Ulcerative Colitis
EEN	Some benefit for inducing remission; Difficult to follow long-term;	Insufficient data.
Partial enteral nutrition	Some benefit for inducing remission; Similar in efficacy as EEN. More palatable than EEN.	Sparse data did not show benefit over habitual diets.
Crohn’s Disease Exclusion Diet	Some benefit for sustaining remission, at least in short term;	Not applicable.
Mediterranean diet	Some benefit for inducing remission; Similar in efficacy as Specific Carbohydrate Diet; One small RCT (n = 24) found Crohn’s disease exclusion diet to more effective than the Mediterranean diet for inducing remission [83].	One small RCT (n = 17) comparing the Mediterranean diet and Specific Carbohydrate Diet was terminated early due to lack of efficacy [88].
Specific Carbohydrate Diet	Similar in efficacy as Mediterranean diet; No published comparison with habitual control diet.	One small RCT (n = 17) comparing the Mediterranean diet and Specific Carbohydrate Diet was terminated early due to lack of efficacy [88].
Low FODMAP diet	Some benefit for reducing IBS-like symptoms with quiescent IBD; Possible benefit for reducing disease activity scores and calprotectin.	Some benefit for reducing IBS-like symptoms with quiescent IBD.
Whole-food diet	One small RCT (n = 28) showed potential benefit for inducing remission [95]. One RCT with 83 pediatric patients found comparable achievement of clinical remission and normalization of CRP and calprotectin [96].	Insufficient data.

Abbreviations: CRP, C-reactive protein; EEN, exclusive enteral nutrition; FODMAP, fermentable oligo-, di-, monosaccharides, and polyols; IBD, inflammatory bowel disease; IBS, irritable bowel syndrome; RCT, randomized controlled trial.

**Table 5:** Characteristics of foods of different particle sizes.

Particle Size	Characteristics	Examples
Small-particle foods	Easy to mash with a fork into small pieces	Smoothies, soups, mashed or pureed fruits, vegetables, beans/legumes (e.g., mashed avocado, hummus, and pureed sweet potato), and ground meats (e.g., ground beef, turkey, and chicken)
Medium-particle foods	Soft-cooked foods	Vegetables and fruits cooked to fork-tender (e.g., steamed broccoli)
Large-particle foods	Raw, tough, or coarse foods	Raw fruits and vegetables, especially those high in insoluble fiber (e.g., berries, kale, and brussels sprouts), and coarse/tough meats (e.g., steak, and sausage)

**Table 6:** Dietary therapies for short bowel syndrome.

Nutrients	SBS with Intact Colon	SBS with Ostomy
Food choices should promote gastrointestinal tolerance, while considering individual preferences, access, culture, lifestyle, and overall quality of life. Diet may gradually liberalize as intestinal adaptation progresses with symptom reduction.		
Calories	Add an extra 500–1000 calories/day into the diet for hyperphagia.	
Carbohydrates	Add complex starches and fiber (e.g., fruits, starchy/non-starchy vegetables, grains) to meals.	Eat fiber as tolerated at meals. Do smaller portions if needed.
	Consider various textures for fibers to bring in small particle size for tolerance (e.g., blended, mashed, or minced such as polenta versus corn on the cob or creamy nut butter versus crunchy nut butter). Acceptable to have lactose (e.g., milk, yogurt, and hard cheese) up to 20 g/day as tolerated. Adjust portions at meals as needed. Limit concentrated sweets (e.g., cake and candy) to small amounts, not consumed daily, and shared with others.	
Protein	Choose animal protein (e.g., meat, poultry, fish, and eggs) of high biological value as much as possible. Acceptable to consume plant protein (e.g., legumes, quinoa, and soy) as tolerated, especially if vegetarian or vegan. Focus on small particle size as needed, as plant protein also has fiber.	
Fat	Follow a low-fat diet with limited intake of fried, oily, and greasy foods.	Acceptable to eat fat as tolerated in the diet.
Oxalates	Follow a low-oxalate diet (e.g., limit high oxalates of berries, wheat, spinach, potato, and nuts) in those with history of nephrolithiasis and/or current signs and symptoms of low urine output (≤1200 mL/day) Follow a low-fat diet. Sip on ORS (commercial and homemade recipes are available) to support hydration.	No restriction is needed.

Abbreviations: ORS, oral rehydration solution; SBS, short bowel syndrome.

**Table 7:** Summary of dietary therapies for gastrointestinal disorders ^a^.

Diet	Proposed Mechanisms	Clinical Indications	Potential Limitations
Fiber supplementation	Soluble fiber improves stool consistency; Insoluble fiber increases water retention; Fiber is fermented into bioactive metabolites by gut microbes.	IBS; Chronic constipation.	Moderate or low overall certainty of evidence.
Low FODMAP diet	Reduced consumption of fermentable simple carbohydrates that lead to increased fluid retention and bacterial gas production.	IBS; Functional dyspepsia with subtypes of postprandial distress syndrome and bloating.	Risk of nutrient deficiency if not properly guided; Moderate or low overall certainty of evidence.
Mediterranean diet	Reduced exposure to pro-inflammatory food antigens; Increased intake of fiber and polyphenols with anti-inflammatory properties.	Crohn’s disease.	Low or very low overall certainty of evidence.
IgG-based elimination diet	Reduced exposure to immunogenic foods suggested by generation of food-specific IgG.	IBS.	Effect of selective IgG-based food elimination vs. removal of common food triggers (e.g., wheat and milk) is currently unclear; Very low overall certainty of evidence.
1-, 2-, 4-, and 6-food elimination diet	Reduced exposure to immunogenic foods.	Eosinophilic esophagitis.	Moderate or low overall certainty of evidence.
Gluten-free diet	Reduced exposure to gluten antigens.	Celiac disease.	Gluten contamination; High or moderate certainty of evidence.
Exclusive enteral nutrition	Reduced exposure to pro-inflammatory food antigens; Modulation of enteric immune system.	Crohn’s disease; Eosinophilic esophagitis.	Poor palatability; Cost considerations; Reduces gut microbial diversity; Moderate, low, or very low overall certainty of evidence.
Partial enteral nutrition	Reduced exposure to pro-inflammatory food antigens; Modulation of enteric immune system.	Crohn’s disease.	Cost considerations; Low overall certainty of evidence.
Crohn’s Disease Exclusion Diet	Reduced exposure to pro-inflammatory food antigens; Increased intake of fiber and polyphenols with anti-inflammatory properties.	Crohn’s disease.	Low overall certainty of evidence.
Specific Carbohydrate Diet	Emphasis on monosaccharides to facilitate absorption and reduced intake of di- and polysaccharides that are hypothesized to be more poorly absorbed and can lead to bacterial overgrowth.	Crohn’s disease.	Restrictive diet; Requires advanced preparation; Low overall certainty of evidence/
Whole-food diet	Reduced exposure to pro-inflammatory food antigens; Increased intake of fiber and polyphenols with anti-inflammatory properties.	Crohn’s disease; IBS.	Low or very low overall certainty of evidence.

Abbreviations: FODMAP, fermented, oligo-, di-, monosaccharides, and polyols; IBS, irritable bowel syndrome. ^a^ for the clinical indications provided, overall certainty of evidence is based on available research data and general clinical observation of effectiveness.

## Data Availability

No new data were created or analyzed in this study. Data sharing is not applicable to this article.
